# Impact of nucleic acid self-alignment in a strong magnetic field on the interpretation of indirect spin–spin interactions

**DOI:** 10.1007/s10858-015-0005-x

**Published:** 2015-12-19

**Authors:** Andrea Vavřinská, Jiří Zelinka, Jakub Šebera, Vladimír Sychrovský, Radovan Fiala, Rolf Boelens, Vladimír Sklenář, Lukáš Trantírek

**Affiliations:** Bijvoet Center for Biomolecular Research, Utrecht University, Padualaan 8, 3584 CH Utrecht, The Netherlands; Faculty of Science, Masaryk University, Kotlářská 2, 611 37 Brno, Czech Republic; Institute of Organic Chemistry and Biochemistry, Academy of Sciences of the Czech Republic, v.v.i., Flemingovo náměstí 542/2, 166 10 Praha 6, Czech Republic; Institute of Physics, Academy of Sciences of the Czech Republic, v.v.i, Na Slovance 2, 182 21 Prague 8, Czech Republic; Central European Institute of Technology, Masaryk University, Kamenice 753/5, 625 00 Brno, Czech Republic

**Keywords:** Nucleic acid, Self-alignment, Magnetic susceptibility, Scalar coupling, Dipolar coupling, Karplus equation

## Abstract

Heteronuclear and homonuclear direct (D) and indirect (J) spin–spin interactions are important sources of structural information about nucleic acids (NAs). The Hamiltonians for the D and J interactions have the same functional form; thus, the experimentally measured apparent spin–spin coupling constant corresponds to a sum of J and D. In biomolecular NMR studies, it is commonly presumed that the dipolar contributions to Js are effectively canceled due to random molecular tumbling. However, in strong magnetic fields, such as those employed for NMR analysis, the tumbling of NA fragments is anisotropic because the inherent magnetic susceptibility of NAs causes an interaction with the external magnetic field. This motional anisotropy is responsible for non-zero D contributions to Js. Here, we calculated the field-induced D contributions to 33 structurally relevant scalar coupling constants as a function of magnetic field strength, temperature and NA fragment size. We identified two classes of Js, namely ^1^J_CH_ and ^3^J_HH_ couplings, whose quantitative interpretation is notably biased by NA motional anisotropy. For these couplings, the magnetic field-induced dipolar contributions were found to exceed the typical experimental error in J-coupling determinations by a factor of two or more and to produce considerable over- or under-estimations of the J coupling-related torsion angles, especially at magnetic field strengths >12 T and for NA fragments longer than 12 bp. We show that if the non-zero D contributions to J are not properly accounted for, they might cause structural artifacts/bias in NA studies that use solution NMR spectroscopy.

**Electronic supplementary material:**

The online version of this article (doi:10.1007/s10858-015-0005-x) contains supplementary material, which is available to authorized users.

## Introduction

The major sources of structural information from NMR measurements of biomolecules in isotropic solution are nuclear Overhauser enhancements (NOEs), which provide information about short (<5 Å) inter-proton distances, and indirect spin–spin interactions that are characterized by scalar coupling constants (J), which provide information about torsion angles (Roberts 1993; Wijmenga and van Buuren 1998). In addition to these two sources, direct spin–spin interactions (D), known as (residual) dipolar couplings (RDCs), reveal the relative orientations of inter-nuclear vectors with respect to the direction of the external magnetic field. The direct spin–spin interactions can be measured under conditions where the studied molecules are at least partially aligned with respect to the magnetic field. The alignment typically requires supplementation of NMR buffers with some type of alignment media, such as bicelles, nonionic polymers, Pf1 bacteriophages, anisotropically compressed gels or covalent modifications of investigated molecules with paramagnetic tags (Bax and Tjandra 1997; Clore et al. 1998; Rückert and Otting 2000; Sass et al. 2000; Su et al. 2008; Tjandra and Bax 1997; Tycko et al. 2000; Wöhnert et al. 2003; Zweckstetter and Bax 2001).

For proteins, NMR structure determination is predominantly based on inter-proton NOEs. However, the structure determination of nucleic acids, particularly axially symmetric and elongated NA constructs, strongly depends on the use of direct and indirect spin–spin interactions due to the inherently low proton density and the absence of long-range contacts (Zhou et al. 1999).

In contrast to both NOEs and residual dipolar couplings, for which analytical relationships between the respective observable and geometry exist, the interpretation of scalar couplings typically relies on the quantitative relationship between the local geometry and the corresponding scalar coupling, established by means of (empirical) parameterization, i.e., by measurement of Js or calculation of Js using methods of quantum chemistry on a set of model molecules with known geometry. At present, approximately 33 distinct scalar coupling constants can be employed for the conformational analysis of nucleic acids. Specifically, the ^3^J_H1′H2′_, ^3^J_H1′H2″_, ^3^J_H2′H3′_, ^3^J_H2″H3′_, ^3^J_H3′H4′_, ^3^J_H1′C3′_, ^3^J_H4′C2′_, ^3^J_H3′C1′_, ^3^J_H2′C4′_, ^2^J_H2′C1′_, ^2^J_H3′C2′_, ^2^J_H2′C3′_, ^2^J_H3′C4′_, ^1^J_H3′C3′_, and ^1^J_H2′C2′_ couplings and their combinations are well established as good indicators of sugar conformations (Wijmenga and van Buuren 1998). Heteronuclear one-bond (^1^J_C1′H1′_) and three-bond scalar couplings, namely, ^3^J_H1′C2/C4_ and ^3^J_H1′C6/C8_, allow for the determination of the relative orientation of the base with respect to the sugar moiety via a description of the glycosidic torsion angle *χ* (Fonville et al. 2012; Ippel et al. 1996; Munzarova and Sklenar 2003; Trantirek et al. 2002). The use of scalar couplings is particularly important for the characterization of the phosphate backbone of NA, where the quantitative relations are established between the following: ^3^J_C4′P_, ^3^J_H5′P_, ^3^J_H5″P_, and ^4^J_H4′P_ and the torsion angle β; ^3^J_H4′H5′_ and ^3^J_H4′H5″_ and the torsion angle γ; and ^3^J_H3′P_, ^3^J_C2′P′_, and ^3^J_C4′P_ and the torsion angle ε (Roberts 1993; Wijmenga and van Buuren 1998). In addition to their quantitative interpretation in terms of the local structure, the scalar couplings can be used to identify the long-range structural features of nucleic acids. Non-zero values of the ^1h^J_NH_ and ^2h^J_NN_ scalar couplings can be used as direct experimental evidence of a hydrogen bond and as a reporter of the base-pairing pattern (Alkorta et al. 2008). Similarly, non-zero values of ^3^J_PC_ and ^2^J_PH_ across the P–O···H–C link report on the presence of specific structural features of nucleic acids, such as the turn-kink motif (Sychrovský et al. 2006).

Experimentally, J couplings are usually measured from E.COSY-type spectra (Griesinger et al. 1985), from spin-state selective (Meissner et al. 1997a; b), IPAP (Ottiger et al. 1998), quantitative J-correlation experiments (Bax et al. 1994), or from the difference in the peak positions in TROSY and decoupled HSQC spectra (Kontaxis et al. 2000). The Hamiltonians for both the indirect (J) and direct (D) spin–spin interaction have the same functional form

1 $$H = 2\pi A_{IS} I_{Z} S_{Z}$$

where *A*_*IS*_ is either the scalar coupling constant *J*_*IS*_ and/or the dipolar coupling constant *D*_*IS*_. As a consequence, the apparent scalar coupling constant that is observed experimentally in the case of molecular alignment is *J*_*IS*_ + *D*_*IS*_. Therefore, equating the measured values to J couplings is generally incorrect and leads to incorrect structural restraints unless the dipolar contribution is negligible. For diamagnetic proteins, random molecular tumbling effectively cancels the dipolar contributions. However, for nucleic acids, the dipolar contributions arising from the anisotropy of molecular tumbling might be significant because the inherent magnetic susceptibility of NAs causes an interaction with the external magnetic field. This motional anisotropy, the so-called self-alignment, was first mentioned as far back as by Robinson (1961), who showed that nucleic acids in solutions above a certain critical concentration can spontaneously undergo transitions from isotropic liquid to nematic liquid crystalline phases (>50 mg/mL for short DNA fragments) (Iizuka 1978; Iizuka and Kondo 1979; Iizuka and Yang 1977; Senechal et al. 1980; Trohalaki et al. 1984). Years later, numerous experimental studies (Brandes and Kearns 1986; Rill 1986; Rill et al. 1983) investigating the effect of increasing DNA concentrations (up to 300 mg/mL) and fragment lengths (147, 234, and 437 bp) on NMR spectral intensities confirmed this finding. In conventional applications of solution NMR spectroscopy for nucleic acid structure determination, which used short NA fragments (10–25 bp), concentration ranges of 0.5–3 mM, and the magnetic field strengths available at that time (5–14 T), the NA self-alignment was considered negligible.

Nevertheless, the interest in the self-alignment phenomenon was renewed with the availability of NMR spectrometers operating at high-magnetic field strengths, which provided sensitivity and resolution amiable to longer NA fragments (up to 40 bp). Between 2001 and 2004, several groups independently demonstrated that the magnetic susceptibility of nucleic acids is capable of producing sufficient self-alignment in dilute solutions of oligonucleotides of moderate lengths to measure the magnetic field-induced RDCs (fiRDCs) that can be employed for NA structural analysis (Al-Hashimi et al. 2001a, c; Bryce et al. 2004; Kung et al. 1995; van Buuren et al. 2004; Zhang et al. 2003). These works provided an important proof-of-concept and showed that RDCs can be obtained under conditions that do not perturb the studied system by the use of either additives (alignment media) or NA fragment paramagnetic tagging. However, the magnitudes of the RDCs obtained from the self-alignment were several times smaller than those routinely achievable using standard alignment media. The considerable relative errors in measuring small fiRDCs have a negative influence on the quality of NA structure refinement. This limitation and the fact that the determination of the fiRDC requires measurements at least two different magnetic field strengths are the primary reasons why NA self-alignment is not routinely used to characterize nucleic acid structure.

In the past, all studies have focused on the potential of NA self-alignment to measure fiRDCs in a non-invasive manner, and the self-alignment phenomenon has not been studied in detail with respect to the interpretation of scalar couplings. The direct (D) and indirect (J) spin–spin interactions have the same form of Hamiltonian, making them inseparable within a single NMR experiment; thus, the scalar coupled spectra should always be treated as spectra “contaminated” by the dipolar contributions. In some cases, this contamination can severely taint the structure determination process. The aim of this paper is to draw attention to the consequences of NA self-alignment on the interpretation of indirect spin–spin interactions in terms of NA structure and to identify problematic situations where the self-alignment might result in structural artifacts.

## Materials and methods

### Quantum chemical calculations

DFT calculations of magnetic susceptibilities were performed on each nucleic acid base (A, G, C, T, and U) using the B3LYP Exchange Correlation Functional (Becke 1993) as implemented in Gaussian 09, Revision A.02 (Frisch et al. 2009). The starting geometries of the five aforementioned nitrogenous bases correspond to idealized geometries of NA bases (Clowney et al. 1996). Subsequently added hydrogen atoms were optimized at the B3LYP/6-31G** level of theory and included the implicit solvent (CPCM) described within the polarizable continuum model (Miertuš et al. 1981; Miertus and Tomasi 1982). The ensuing GIAO calculations (Cheeseman et al. 1996; Wollinski et al. 1990) of the base *χ* magnetic susceptibility tensors were performed using the Pople triple-zeta-valence basis set 6-311++G(3df,3pd), with multiple polarizations used on all atoms (Ditchfield et al. 1971). The resulting computed nucleobase magnetic susceptibility tensors were expressed in the form of 3 × 3 symmetric matrix that is the sum of an isotropic (zeroth rank) and an anisotropic symmetrical (second rank) tensor.

### Molecular anisotropy of magnetic susceptibility

Three-dimensional Cartesian coordinate models (NA fragments consisting of 12, 24, and 36 bp) were generated for the canonical conformation of the double-helical A-RNA and B-DNA using the 3DNA (Lu 2003) and AMBER 10 Molecular Dynamics Software Package (Case et al. 2008). The sequences of individual fragments are listed in Table S1. The respective molecular magnetic susceptibilities of the model molecules were then calculated from tensor summations of the individual values of the nucleobase-specific magnetic susceptibilities (Bryce et al. 2004). Through an appropriate orthogonal transformation that diagonalizes the molecular magnetic susceptibility tensor into the principal axis frame and through the subsequent subtraction of the isotropic contribution, the anisotropic part of the molecular magnetic susceptibility (AMMS) tensor was obtained. The molecular tensor was described using its three non-degenerate eigenvalues and eigenvectors. The eigenvalues were sorted according to their absolute values as:

2 $$\left| {\chi_{33} -_{iso} } \right| \ge \left| {\chi_{11} - \chi_{iso} } \right| \ge \left| {\chi_{22} - \chi_{iso} } \right|$$

to determine the anisotropy Δ*χ* and rhombicity *R* of the AMMS tensor.

3 $$\varDelta \chi = \chi_{33} - \frac{1}{2}\left( {\chi_{11} + \chi_{22} } \right)$$

4 $$R = {{\left( {\chi_{22} - \chi_{11} } \right)} \mathord{\left/ {\vphantom {{\left( {\chi_{22} - \chi_{11} } \right)} {\varDelta \chi }}} \right. \kern-0pt} {\varDelta \chi }}$$

The fiRDCs were calculated as a function of the AMMS tensor according to the following equation:

5 $$fiRDC(Hz) = - \left[ {\frac{{\mu_{0} (B_{0} )^{2} \varDelta \chi S\gamma_{I} \gamma_{S} h}}{{240\pi^{3} kTr_{IS}^{3} }}} \right]\left[ {\left( {3\cos^{2} \theta - 1} \right)\frac{3}{2}R\sin^{2} \theta \cos 2\phi } \right]$$

where *S* is the generalized order parameter, *γ*_*I*_, *γ*_*S*_ are the magnetogyric ratios of nuclei *I* and *S*, respectively, and Δχ and *R* are the anisotropy and rhombicity, respectively, of the AMMS tensor. *r*_*IS*_ is the internuclear distance, and *θ* and *ϕ* are polar coordinates describing the orientation of the internuclear I-S vector in the principal axis system of the molecular magnetic susceptibility tensor.

#### Note

Experimental validation of the reconstruction approach based on nucleobase-specific magnetic susceptibilities can be found in Bryce et al. (2004).

## Results and discussion

Unlike the RDCs induced by orienting media that are evaluated by comparing the spectra measured in isotropic and orienting solutions, the magnetic field-induced dipolar couplings can never be completely switched off. If not taken into account, the fiRDCs might become a source of systematic errors. To identify the scalar couplings whose quantitative interpretation is potentially biased by NA self-alignment we simulated the magnetic field-induced dipolar contributions to all currently used J-coupling constants for NA structural analysis as a function of the strength of the external magnetic field (9.4, 11.8, 22.3, and 28.1 T), the temperature (278, 293, and 308 K), and the length of the NA fragment (12, 24, and 36 bp) for the two most common nucleic acid motifs, namely A-DNA (A-RNA) and B-DNA. For 15 of 33 calculated Js, the magnetic field-induced RDC contributions were found to exceed the typical experimental error in J-coupling determinations by a factor of two or more (Tjandra et al. 1996; Wang and Bax 1996; Yao et al. 2009). These J couplings are potential sources of interpretational bias, and they can be formally divided into two different categories: (1) ^1^J_CH_ and (2) ^3^J_HH_. The effect of self-alignment on the quantitative interpretation of these J couplings in terms of structure was analyzed in detail (vide infra). For a complete overview of the simulated RDC contributions, see Supplementary Information—Tables S2 and S3.

### Effect of self-alignment on the interpretation of ^1^J_CH_

**Fig. 1:**
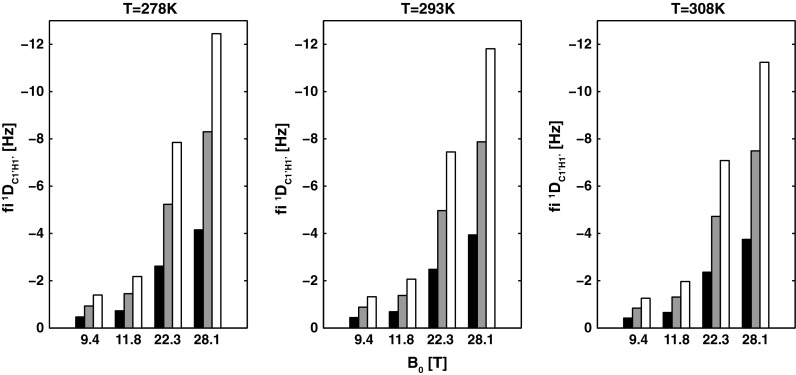
Calculated fi^1^D_C1′H1′_ values for residue G10 in canonical B-DNA. Each fi^1^D_C1′H1′_ value was computed at four magnetic field strengths B_0_ (9.4, 11.8, 22.3, and 28.1 T), three different temperatures T (278, 293, and 308 K) and three different fragment lengths (12, 24, and 36 bp). The 12 bp fragment is indicated in *black*, the 24 bp fragment in *grey*, and the 36 bp fragment in *white*

The magnitude of magnetic field-induced residual dipolar couplings is inversely proportional (r^−3^) to the distance of interacting nuclei; thus, it is not surprising that one-bond ^1^J_CH_ couplings display some of the largest magnetic field-induced dipolar contributions (Supplementary Information—Tables S2 and S3). Figure 1 shows the result of the simulation of the dipolar contribution to the structurally important ^1^J_C1′H1′_, which provides information about the conformation of the glycosidic torsion angle (χ); this angle describes the relative orientations of NA bases and sugar moieties in the model B-DNA fragment. The simulation was performed as a function of temperature, magnetic field strength, and length of the investigated NA fragment.

**Fig. 2:**
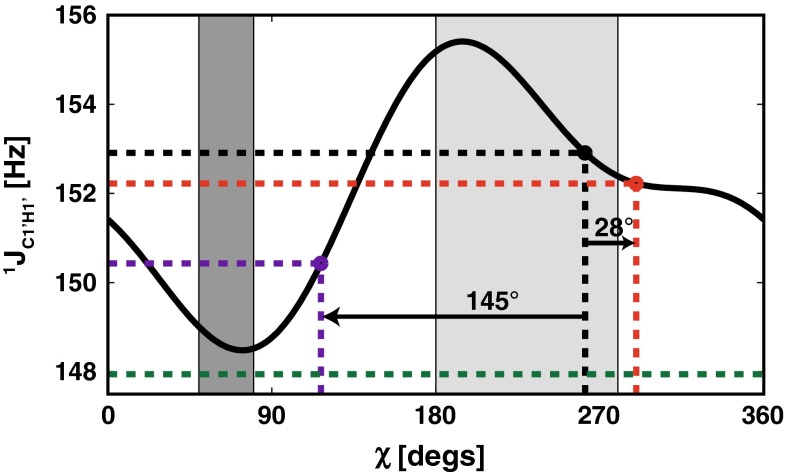
The Karplus curve for ^1^J_C1′H1′_ (parameterization according to Munzarova and Sklenar 2003). The *black filled circle* corresponds to the true ^1^J_C1′H1′_ coupling (152.9 Hz) expected for residue G10 (*χ* = 262°) in the 12 bp canonical B-DNA. The *red and purple filled circles* indicate the apparent ^1^J_C1′H1′_ values that correspond to the sum of the true ^1^J_C1′H1′_ values and the dipolar contributions resulting from the DNA fragment self-alignment at 293 K and at magnetic field strengths of 11.8 T (−0.7 Hz) and 22.3 T (−2.5 Hz), respectively. The *arrows* indicate the errors in the interpretation of the apparent ^1^J_C1′H1′_ value due to dipolar contributions. For the 24 bp fragment at 293 K and a magnetic field strength of 22.3 T, the dipolar contribution reaches ~5 Hz; thus, the apparent ^1^J_C1′H1′_ value falls outside the ranges defined by the Karplus curve (*green dashed line*). The light grey area indicates the boundaries typical for the *anti* conformation of χ (180°–280°). The *dark grey area* marks the typical boundaries for the *syn* conformation of χ (50°–80°)

As shown in Fig. 1, the contribution of fi^1^D_C1′H1′_ to the apparent ^1^J_C1′H1′_ primarily depends on the magnetic field strength and length of the nucleic acid fragment, whereas the temperature dependence has a marginal effect (<10 % within the range from 5 to 35 °C). Our calculations show that for the small model B-DNA fragment (12 bp length) and at the low magnetic field strength of 11.8 T, the fi^1^D_C1′H1′_ contribution to the apparent ^1^J_C1′H1′_ for the residue G10 reaches −0.7 Hz. As shown in Fig. 2, if not properly accounted for during the interpretation of the apparent ^1^J_C1′H1′_, even this relatively small contribution will lead to an approximately 28° overestimation of the χ torsion angle from the established Karplus equation. However, in this case, the corresponding structural error is still within the typical error bounds for the torsion angle restraints derived from the Karplus equation (±30°). Importantly, such a small fiRDC contribution does not alter the qualitative interpretation of the χ torsion angle, which is correctly assigned to the *anti* conformation. However, for the same-sized fragment at B_0_ = 22.3 T, the corresponding fi^1^D_C1′H1′_ contribution reaches −2.5 Hz (Fig. 1; Supplementary Information—Table S2). In this case, the appropriate Karplus equation will incorrectly assign the χ torsion angle to the region between the *anti* and *syn* conformations. For a moderate sized NA fragment that is 24 bp long at B_0_ = 22.3 T, the fi^1^D_C1′H1′_ exceeds −4.9 Hz. In this case, the apparent J coupling would fall outside the ranges covered by the corresponding Karplus equation. The same situation will apply to any NA fragments that have a comparable or higher anisotropy of the magnetic susceptibility at magnetic fields equal to or exceeding 22.3 T. With the upcoming generation of NMR spectrometers operating at magnetic fields reaching up to 28 T and providing sensitivity and resolution suitable for structural investigations of large NA systems, the interpretation bias of the apparent ^1^J_C1′H1′_ stemming from the fiRDC contribution will be even more pronounced. However, at the practical level, these large fi^1^D_C1′H1′_ contributions are unlikely to be overlooked because the measured apparent Js will fall out of the range defined by the established Karplus equation. In these cases, the apparent ^1^J_C1′H1′_ value that is uncorrected for the fi^1^D_C1′H1′_ will produce violations with other, magnetic field-independent NMR restraints, such as those from H1′–H6/8 NOEs. However, for NA fragments of moderate size (20–50 nt) measured at moderate-to-high magnetic fields (14–17 T), the fi^1^D_C1′H1′_ contribution might be easily unnoticed because the direct structural interpretation of the apparent ^1^J_C1′H1′_ will still lie within the ranges indicated by loosely defined H1′–H6/8 NOEs. A direct interpretation of the apparent ^1^J_C1′H1′_ (without correction for fi^1^D_C1′H1′_) will provide a correct assignment of the χ torsion angle, e.g., into the anti-periplanar region (Fig. 2); however, in quantitative terms, an under- or over-estimation of the χ for a single base by more than 20° is expected to propagate through the NA helix via the van der Waals term describing stacking and base separation and inter-base NOEs in the rMD, which is typically employed for NMR restraint-based structure determination.

For other structurally important ^1^J_CH_s, such as ^1^J_C3′H3′_ and ^1^J_C2′H2′_ that provide information about the conformation of the sugar ring or ^1^J_H5′C5′_ and ^1^J_H5″C5′_, which are used for stereospecific assignment of the H5′ and H5″ resonances, the situation is analogous to the ^1^J_C1′H1′_. In general, the absolute values of the corresponding fiRDCs increase with increasing magnetic field strength as well as with increasing nucleic acid fragment sizes (Supplementary Information—Tables S2 and S3). The interpretation of ^1^J_C3′H3′_ and ^1^J_C2′H2′_ is based on the observation that for N-type sugars, the ^1^J_C2′H2′_ and the ^1^J_C3′H3′_ values are approximately 8 Hz higher and lower, respectively, than their values in S-type sugars (Ippel et al. 1996). For ^1^J_C2′H2′_ and ^1^J_C3′H3′_ in both N-type and S-type sugars, the corresponding fiRDCs are significant, and they have comparable magnitudes and signs (Supplementary Information—Tables S2 and S3). Consequently, the fiRDCs for those Js do not change their relative differences and do not affect their structural interpretation. The situation with the fi^1^D_C5′H5′/H5″_ demonstrates that fiRDC might even, in certain cases, facilitate the NA structure determination process. The ^1^J_H5′C5′_ and ^1^J_H5″C5′_ values are being used for the stereospecific assignment of H5′ and H5″. The assignment is based on fact that ^1^J_H5′C5′_ is generally larger than ^1^J_H5″C5′_ (Ippel et al. 1996). The presence of the fi^1^D_C5′H5′_ and fi^1^D_C5′H5″_ contributions makes the difference between the ^1^J_H5′C5′_ and ^1^J_H5″C5′_ values even more pronounced because the absolute magnitudes of fi^1^D_H5′C5′_ and fi^1^D_H5″C5′_ are comparable, whereas their signs differ (Supplementary Information—Tables S2 and S3). Taken together, these results show the following: For fi^1^D_C1′H1′_, disregarding the dipolar contribution is always connected with interpretational bias. In contrast, the fiRDC contributions to ^1^J_C3′H3′_ and ^1^J_C2′H2′_ as well as those to ^1^J_H5′C5′_ and ^1^J_H5″C5′_ is not expected to impair the corresponding apparent ^1^J_C–H_s interpretation.

### Effect of self-alignment on the interpretation of ^3^J_HH_

**Fig. 3:**
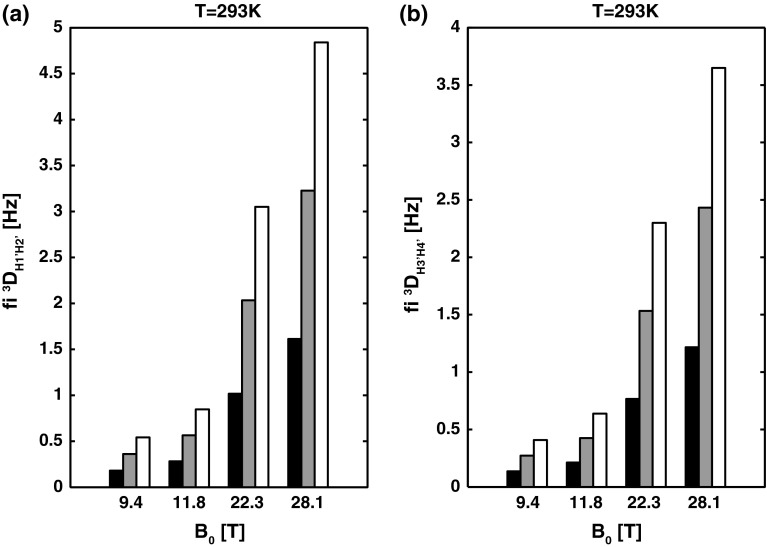
Calculated fi^3^D_H1′-H2′_ for residues C3 (**a**) and G10 (**b**) in canonical A-RNA and B-DNA, respectively. The dipolar coupling values are computed at 293 K, at four magnetic field strengths B_0_ (9.4, 11.8, 22.3, and 28.1 T), and three different NA fragment lengths (12, 24, and 36 bp). The 12 bp fragment is indicated in *black*, the 24 bp fragment in *grey*, and the 36 bp fragment in *white*

**Fig. 4:**
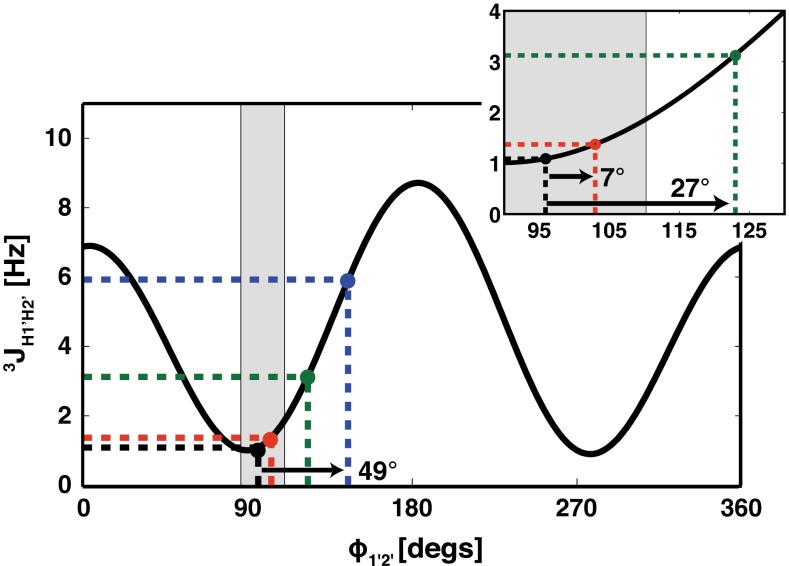
The Karplus curve for ^3^J_H1′H2′_ (parameterization according to Roberts Munzarova and Sklenar 2003). The *black filled circle* corresponds to the true ^3^J_H1′-H2′_ coupling (1.1 Hz) expected for residue C3 (torsion angle *ϕ*_1′2′_ = 96°) in the 12 bp canonical A-RNA. The *red, green, and blue filled circles* indicate the apparent ^3^J_H1′H2′_ values that correspond to the sum of true ^3^J_H1′H2′_ values and the dipolar contributions resulting from the self-alignment of the 12, 24, and 36 bp RNA fragments at 293 K and at a magnetic field strength of 11.8 T (0.3 Hz), 22.8 T (2 Hz) and 28.1 T (4 Hz), respectively. The *arrows* in the inset indicate the errors in the interpretation of the apparent ^3^J_H1′H2′_ value due to dipolar contributions. The *light grey* area indicates the *ϕ*_1′2′_ torsion angle boundaries typical for the C3′-endo conformation (86°–110°) for P^N^ = <0°,36°> and *φ*_*m*_ = <35°,42°> (Roberts 1993)

**Fig. 5:**
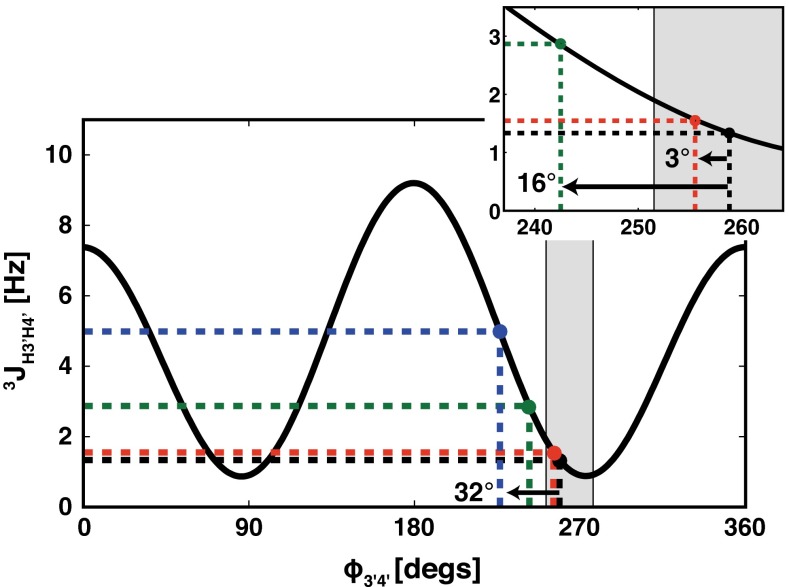
The Karplus curve for ^3^J_H3′H4′_ (parameterization according to Roberts (Munzarova and Sklenar 2003)). The *black filled circle* corresponds to the true ^3^J_H3′H4′_ coupling (1.3 Hz) expected for residue G10 (torsion angle *ϕ*_3′4′_ = 258.8°) in the 12 bp canonical B-DNA. The *red, green, and blue filled circles* indicate the apparent ^3^J_H3′H4′_ values that correspond to the sum of the true ^3^J_H3′H4′_ values and the dipolar contributions resulting from the self-alignment of the 12, 24, and 36 bp DNA fragments at 293 K and at magnetic field strengths of 11.8 T (0.2 Hz), 22.8 T (1.5 Hz) and 28.1 T (3.6 Hz), respectively. The *arrows* in the inset indicate the errors in the interpretation of the apparent ^3^J_H1′-H2′_ due to the dipolar contributions. The *light grey* area indicates the typical *ϕ*_3′4′_ torsion angle boundaries for the C2′-endo conformation (86°–110°) for P^N^ = <0°,36°> and *φ*_*m*_ = <35°,42°> (Roberts 1993)

A second group of J couplings that are notably affected by fiRDC contributions are the three-bond proton–proton scalar couplings (^3^J_HH_). Although the inter-proton distance between scalar coupled protons over three bonds is considerably longer than that of the one-bond C–H, the fiRDC contribution to ^3^J_HH_ arises due to the large value of the product of the proton gyromagnetic constants (see Eq. 5). Nonetheless, compared to fi^1^D_CH_, the fi^3^D_HH_ values are notably smaller, ranging from |0.1| to |3| Hz for fragments between 12 and 36 bp and magnetic fields strength of 9.4–22.3 T (Supplementary Information—Tables S2 and S3). Among the ^3^J_HH_s commonly used for NA structure determination, two ^3^J_HH_s are particularly useful in the determination of the conformation of the sugar ring, namely ^3^J_H1′H2′_ and ^3^J_H3′H4′_. Our calculations indicate that for small double helical NA fragments (~12 bp) investigated at low magnetic field strengths (<12 T), neither fi^3^D_H1′H2′_ nor fi^3^D_H3′H4′_ (~0.3 Hz) biases the qualitative interpretation of the apparent ^3^J_H1′H2′_ and ^3^J_H3′H4′_ values in terms of the sugar pucker conformation (Figs. 4, 5). However, the calculations show that the fi^3^D_H1′H2′_ contribution reaches ~2 Hz (Fig. 3a, Supplementary Information—Table S2) for the 24 bp A-RNA fragment at 22.3 T. Analysis of the effect of the fi^3^D_H1′H2′_ value on interpretation of the apparent ^3^J_H1′H2′_ shows that such fiRDC will produce a 27° error in the torsion angle ϕ_H1′H2′_ (Fig. 4). At a field strength of 28. T, the error in the torsion angle ϕ_H1′H2′_ due to an fiRDC contribution reaching ~3.2 Hz (Supplementary Information—Table S2) for 36 bp A-RNA will reach almost 50° (Fig. 4).

For the apparent ^3^J_H3′H4′_, our calculation indicates that the corresponding fi^3^D_H3′H4′_ reaches ~1.5 Hz (Fig. 3b, Supplementary Information—Table S3) for the 24 bp B-DNA fragment at 22.3 T. Analysis of the effect of the fi^3^D_H3′H4′_ on the interpretation of the apparent ^3^J_H3′H4′_ shows that such fiRDC will produce a 16° error in the pseudo-torsion angle ϕ_H3′H4′_ (Fig. 5). The error increases with increases in the size of the NA fragment (36 bp) and increases in the strength of the magnetic field (28.1 T) up to 32° (Fig. 5).

### ^2/3/4^J_CH_ and ^2/3/4^J_HP_ fiRDC

At currently used magnetic field strengths, the fiRDC contributions to the other commonly used Js in the NMR structure determination of NAs, namely 2- to 4-bond J_CH_s and J_HP_s, are generally below the experimental error and well-within the error bounds used for the J interpretation of nucleic acid structure (Supplementary Information—Tables S2 and S3). However, at the magnetic fields corresponding to the current state-of-the-art 1 GHz spectrometers and the upcoming generation of 1.2 GHz spectrometers, the fiRDC contribution to a number of these structurally important Js become notable and should be taken into consideration during Js structural interpretations (Supplementary Information—Tables S2 and S3). In situations when significant errors are suspected, the pure J value should be determined from measurements at two or more magnetic field strengths (Bryce et al. 2004).

6 $$\begin{aligned} \left\{ {\left( {{}^{n}J_{IS} + {}^{n}D_{IS} } \right)^{high} - \left( {{}^{n}J_{IS} + {}^{n}D_{IS} } \right)^{low} } \right\}\left[ {\frac{{\left( {B_{0}^{high} } \right)}}{{\left( {B_{0}^{high} } \right)^{2} - \left( {B_{0}^{low} } \right)^{2} }}} \right] \hfill \\ = - \left[ {\frac{{\mu_{0} (B_{0} )^{2} \varDelta \chi S\gamma_{I} \gamma_{S} h}}{{240\pi^{3} kTr_{IS}^{3} }}} \right]\left[ {\left( {3\cos^{2} \theta - 1} \right) + \frac{3}{2}R\sin^{2} \theta \cos 2\phi } \right] \hfill \\ \end{aligned}$$

where B^high^ and B^low^ corresponds to high and low magnetic field strengths, respectively. (J + D)^high^ and (J + D)^low^ correspond to the apparent J extracted from the measurements at low and high magnetic field strengths, respectively. Such measurements, however, impose requirements on the available instrumentation and experimental time. On the other hand, at ultra-high magnetic fields, the extracted fiRDC values are expected to become an important source of long-range structural restraints under non-invasive conditions. Avoiding the use of alignment media is particularly important for DNA, which displays notable sensitivity towards non-specific physical–chemical factors, such as ion strength, ion type, molecular crowding, water activity and/or the presence of small osmolytes (Fiala et al. 2011; Hansel et al. 2011).

In the process of J coupling interpretation the errors from fiRDCs, which are the subject of the present study, will add to the other known errors such as those due to neglect of J averaging by internal motion and those due to passive spin-relaxation, referred to as spin-flip(s) (Harbison 1993; Bruschweiler and Case 1994; Vogeli et al. 2008). The spin-flip phenomenon comes for the interference between J-coupling and cross-relaxation and its primary effect is reduction in apparent J. As the effect of spin flip is indirectly proportional to T_1_, the respective error is most significant for small NA fragments (studied at low magnetic fields) and decreases rapidly with the molecular size (particularly when studied at high magnetic fields). For example, the error in ^3^J_HH_ coupling due to spin-flip reaches up to 1 Hz for 12–14 bp NA fragment while the corresponding error will be smaller than 0.1 Hz for 36 bp NA fragment (Harbison 1993). Similarly to the error due to spin flip, the averaging of J by internal motion leads to reduction in apparent J. For structured parts of NA, the errors due to the neglect of motional J averaging are expected to be smaller than 1 Hz (Bruschweiler and Case 1994; Trantirek et al. 2002; Vokacova et al. 2009). Altogether, the neglect of fiRDC contribution appears to be one of the most significant sources of bias in quantitative interpretation of J couplings, especially for medium to larger size nucleotides studied in high magnetic fields.

## Conclusion

The fiRDCs can serve as both an important source of information on the structure and dynamics as well as, if not properly accounted for, a source of structural artifacts/bias in the solution NMR spectroscopy of nucleic acids. Although the usefulness of the fiRDCs for the structural characterization of nucleic acids and their complexes was demonstrated by number of studies (Al-Hashimi 2013; Al-Hashimi et al. 2001b; Zhang and Al-Hashimi 2008), the contributions from fiRDCs to apparent J couplings are among the current most overlooked sources of artifacts in the structure determination of nucleic acids. With recent advances in NMR instrumentation as well as in the automation of the nucleic acid structure determination process, NMR spectroscopy is becoming accessible to a growing community of non-expert users employing pre-programmed “black-box” routines for the interpretation of acquired primary NMR data. The corrections for the fiRDCs are not routinely implemented in the current generation of programs for automated nucleic acid structure determination; thus, an unquestioning use of these programs might adversely affect the quality of NA structures derived from solution NMR data. The situation is expected to worsen in the future with the upcoming generations of NMR spectrometers operating at magnetic fields of up to 28 T, where the fiRDC contributions to apparent J couplings will in many cases become comparable to or even exceed the modulation of the J couplings due to the local conformation. At the currently commonly available magnetic fields (11–17 T), disregarding the fiRDC contributions when interpreting J couplings could in principle be tolerated for the production of low-resolution structural models based on semi-quantitative NMR data; however, properly accounting for fiRDCs appears to be essential for the production of precise and accurate nucleic acid structures. Moreover, accounting for fiRDC contributions is particularly important in applications involving empirical (re)-parameterizations of Karplus equations. Studies that correlate experimental J couplings with the J couplings from quantum chemical calculations, especially studies aiming at benchmarking the calculation methods, must pay particular attention to the fiRDC-induced contamination of J.

## Electronic supplementary material

Below is the link to the electronic supplementary material.

Supplementary material 1 (PDF 307 kb)
