# Supplementary material for: Impact of nucleic acid self-alignment in a strong magnetic field on the interpretation of indirect spin–spin interactions
Source: J Biomol NMR. 2015 Dec 19;64:53–62. doi: 10.1007/s10858-015-0005-x (PMC4742510; doi:10.1007/s10858-015-0005-x)
Supplement: Supplementary file 1 — Supplementary material 1 (PDF 307 kb) [file 10858_2015_5_MOESM1_ESM.pdf]

Supplementary Information

Impact of nucleic acid self-alignment in a strong magnetic field on the interpretation of indirect spin-spin interactions.

Andrea Vavrinska[1], Jiri Zelinka[2], Jakub Sebera[3,4], Vladimir Sychrovsky[4], Radovan Fiala[5], Rolf Boelens[1], Vladimir Sklenar[5], Lukas Trantirek[5]*

[1] Bijvoet Centre for Biomolecular Research, Utrecht University, Padualaan 8, 3584 CH Utrecht, The Netherlands

[2] Faculty of Science, Masaryk University, Kotlarska 2, 602 00 Brno, Czech Republic

[3] Institute of Physics, Academy of Sciences of the Czech Republic, v.v.i, Na Slovance 2, CZ-182 21 Prague 8, Czech Republic

[4] Institute of Organic Chemistry and Biochemistry, Academy of Sciences of the Czech Republic, Flemingovo namesti 2, 111 50 Prague, Czech Republic

[5] Central European Institute of Technology – Masaryk University, Kamenice 735/5, 625 00 Brno, Czech Republic

**Corresponding author*: lukas.trantirek@ceitec.muni.cz

Table S1: List of sequences used to construction of of 12, 24, and 36 bp 3D models of canonical A-RNA and B-DNA.

Model	Sequence 5'->3'
**A-RNA**	
12 bp	$r(CGCGAAUUCGCG)_2$
24 bp	$r(CGCGAAUUCGCGCGCGAAUUCGCG)_2$
36 bp	$r(CGCGAAUUCGCGCGCGAAUUCGCGCGCGAAUUCGCG)_2$
**B-DNA**	
12 bp	$d(CGCGAATTCGCG)_2$
24 bp	$d(CGCGAATTCGCGCGCGAATTCGCG)_2$
36 bp	$d(CGCGAATTCGCGCGCGAATTCGCGCGCGAATTCGCG)_2$

Table S2: Calculated magnetic field induced dipolar couplings $^{1}D_{CH}$ [Hz] (A), $^{2}D_{CH}$ (B), $^{3}D_{HH}$ (C), $^{3}D_{HC}$ (D), $^{3}D_{HP/CP}$ (E) for residue Cyt3 in canonical 12, 24, and 36 bp A-RNA as a function of magnetic field strength B_0 (9.4T, 11.75T, 22.31T and 28.1T) and temperature T (278.15K, 293.15K, 308.15K).

A)

	^{1}D C1′-H1′	^{1}D C2′-H2′	^{1}D C3′-H3′	^{1}D C5′-H5′	^{1}D C5′-H5″
B_0=9.4T; T=278.15K bp=12	0.1903	-0.353	0.3788	0.3902	0.6011
B_0=9.4T; T=278.15K bp=24	0.3806	-0.706	0.7676	0.7804	1.2022
B_0=9.4T; T=278.15K bp=36	0.5709	-1.059	1.1364	1.1706	1.8033
B_0=9.4T; T=293.15K bp=12	0.1806	-0.3349	-0.3594	0.3703	0.5703
B_0=9.4T; T=293.15K bp=24	0.3612	-0.6698	-0.7188	0.7406	1.1406
B_0=9.4T; T=293.15K bp=36	0.5418	-1.0047	-1.0782	1.1109	1.7109
B_0=9.4T; T=308.15K bp=12	0.1718	-0.3186	-0.3419	0.3523	0.5425
B_0=9.4T; T=308.15K bp=24	0.3436	-0.6372	-0.6838	0.7046	1.085
B_0=9.4T; T=308.15K bp=36	0.5154	-0.9558	-1.0257	1.0569	1.6275
B_0=11.75T; T=278.15K bp=12	0.2974	-0.5515	-0.5918	0.6098	0.9392
B_0=11.75T; T=278.15K bp=24	0.5948	-1.103	-1.1836	1.2196	1.8784
B_0=11.75T; T=278.15K bp=36	0.8922	-1.6545	-1.7754	1.8294	2.8176
B_0=11.75T; T=293.15K bp=12	0.2821	-0.5233	-0.5615	0.5786	0.8911
B_0=11.75T; T=293.15K bp=24	0.5642	-1.4066	-1.123	1.1572	1.7822

B_0=11.75T; T=293.15K bp=36	0.8463	-1.5699	-1.6845	1.7358	2.6733
B_0=11.75T; T=308.15K bp=12	0.2684	-0.4978	-0.5342	0.5504	0.8477
B_0=11.75T; T=308.15K bp=24	0.5368	-0.9956	-1.0684	1.1008	1.6954
B_0=11.75T; T=308.15K bp=36	0.8052	-1.4934	-1.6026	1.6512	2.5431
B_0=22.31T; T=278.15K bp=12	1.072	-1.9883	-2.1336	2.1983	3.3858
B_0=22.31T; T=278.15K bp=24	2.144	-3.9766	-4.2672	4.3966	6.7716
B_0=22.31T; T=278.15K bp=36	3.216	-5.9649	-6.4008	6.5949	10.1574
B_0=22.31T; T=293.15K bp=12	1.0172	-1.8866	-2.0244	2.0858	3.2126
B_0=22.31T; T=293.15K bp=24	2.0344	-3.7732	-4.0488	4.1716	6.4252
B_0=22.31T; T=293.15K bp=36	3.0516	-5.6598	-6.0732	6.2574	9.6378

	^{1}D C1′-H1′	^{1}D C2′-H2′	^{1}D C3′-H3′	^{1}D C5′-H5′	^{1}D C5′-H5″
B_0=22.31T; T=308.15K bp=12	0.9677	-1.7948	-1.9259	1.9843	3.0562
B_0=22.31T; T=308.15K bp=24	1.9354	-3.5896	-3.8518	3.9686	6.1124
B_0=22.31T; T=308.15K bp=36	2.9031	-5.3844	-5.7777	5.9529	9.1686
B_0=28.1T; T=278.15K bp=12	1.7007	-3.1543	-3.3848	3.4874	5.3713
B_0=28.1T; T=278.15K bp=24	3.4014	-6.3086	-6.7696	6.9748	10.7426
B_0=28.1T; T=278.15K bp=36	5.1021	-9.4629	-10.1544	10.4622	16.1139
B_0=28.1T; T=293.15K bp=12	1.6137	2.9929	-3.2116	3.3089	5.0965
B_0=28.1T; T=293.15K bp=24	3.2274	-5.9858	-6.4232	6.6178	10.193
B_0=28.1T; T=293.15K bp=36	4.8411	-8.9787	-9.6348	9.9267	15.2895
B_0=28.1T; T=308.15K bp=12	1.5351	-2.8472	-3.0553	3.1479	4.8484
B_0=28.1T; T=308.15K bp=24	3.0702	-5.6944	-6.1106	6.2958	9.6968
B_0=28.1T; T=308.15K bp=36	4.6053	-8.5416	-9.1659	9.4437	14.5452

B)

	^{2}D C1′-H2′	^{2}D C2′-H3′	^{2}D C3′-H2′	^{2}D C4′-H3′
B_0=9.4T; T=278.15K bp=12	0.0496	-0.0068	-0.0538	-0.0024
B_0=9.4T; T=278.15K bp=24	0.0992	-0.0136	-0.1076	-0.0048
B_0=9.4T; T=278.15K bp=36	0.1488	-0.0204	-0.1614	-0.0072

B_0=9.4T; T=293.15K bp=12	0.47	-0.0065	-0.0511	-0.0023
B_0=9.4T; T=293.15K bp=24	0.094	-0.013	-0.1022	-0.0046
B_0=9.4T; T=293.15K bp=36	0.141	-0.0195	-0.1533	-0.0069
B_0=9.4T; T=308.15K bp=12	0.0447	-0.0062	-0.0486	-0.0022
B_0=9.4T; T=308.15K bp=24	0.0894	-0.0124	-0.0972	-0.044
B_0=9.4T; T=308.15K bp=36	0.1341	-0.0186	-0.1458	-0.066
B_0=11.75T; T=278.15K bp=12	0.0775	-0.0106	-0.0841	-0.0038
B_0=11.75T; T=278.15K bp=24	0.155	-0.0212	-0.1682	-0.0076
B_0=11.75T; T=278.15K bp=36	0.2325	-0.0318	-0.2523	-0.0114
B_0=11.75T; T=293.15K bp=12	0.0735	-0.0101	-0.0798	-0.0036
B_0=11.75T; T=293.15K bp=24	0.147	-0.0202	-0.1596	-0.0072
B_0=11.75T; T=293.15K bp=36	0.2205	-0.0303	-0.2394	-0.0108
B_0=11.75T; T=308.15K bp=12	0.0699	-0.0096	-0.0759	-0.0034
B_0=11.75T; T=308.15K bp=24	0.1398	-0.0192	-0.1518	-0.0068
B_0=11.75T; T=308.15K bp=36	0.2097	-0.0288	-0.2277	-0.0102
B_0=22.31T; T=278.15K bp=12	0.2792	-0.384	-0.3033	-0.0136
B_0=22.31T; T=278.15K bp=24	0.5584	-0.768	-0.06066	-0.0272
B_0=22.31T; T=278.15K bp=36	0.8376	-1.152	-0.9099	-0.0408
B_0=22.31T; T=293.15K bp=12	0.2649	-0.0364	-0.2878	-0.0129
B_0=22.31T; T=293.15K bp=24	0.5298	-0.0728	-0.5756	-0.0258
B_0=22.31T; T=293.15K bp=36	0.7947	-0.1092	-0.8634	-0.0387
B_0=22.31T; T=308.15K bp=12	0.252	-0.0374	-0.2738	-0.0123
B_0=22.31T; T=308.15K bp=24	0.504	-0.0748	-0.5476	-0.0246
B_0=22.31T; T=308.15K bp=36	0.756	-0.1122	-0.8214	-0.0369
	^{2}D C1′-H2′	^{2}D C2′-H3′	^{2}D C3′-H2′	^{2}D C4′-H3′
B_0=28.1T; T=278.15K bp=12	0.443	-0.0609	-0.4811	-0.0216
B_0=28.1T; T=278.15K bp=24	0.886	-0.1218	-0.9622	-0.0432
B_0=28.1T; T=278.15K bp=36	1.329	-0.1827	-1.4433	-0.0648
B_0=28.1T; T=293.15K bp=12	0.4203	-0.0578	-0.4565	-0.0205
B_0=28.1T; T=293.15K	0.8406	-0.1156	-0.913	-0.041

bp=24				
B_0=28.1T; T=293.15K bp=36	1.2609	-0.1734	-1.3695	-0.0615
B_0=28.1T; T=308.15K bp=12	0.3998	-0.55	-0.4343	-0.0195
B_0=28.1T; T=308.15K bp=24	0.7996	-0.11	-0.8686	-0.039
B_0=28.1T; T=308.15K bp=36	1.1994	-0.165	-1.3029	-0.0585

C)

	^{3}D H1′-H2′	^{3}D H2′-H3′	^{3}D H3′-H4′	^{3}D H4′-H5′	^{3}D H4′-H5″
B_0=9.4T; T=278.15K bp=12	0.1903	-0.1181	-0.0582	-0.1338	-0.0856
B_0=9.4T; T=278.15K bp=24	0.3806	-0.2362	-0.1164	-0.2676	-0.1712
B_0=9.4T; T=278.15K bp=36	0.5709	-0.3543	-0.1746	-0.4014	-0.2568
B_0=9.4T; T=293.15K bp=12	0.1805	-0.112	-0.0553	-0.127	-0.0813
B_0=9.4T; T=293.15K bp=24	0.361	-0.0224	-0.1106	-0.254	-0.1626
B_0=9.4T; T=293.15K bp=36	0.5415	-0.336	-0.1659	-0.381	-0.2439
B_0=9.4T; T=308.15K bp=12	0.1717	-0.1066	-0.0526	-0.1208	-0.0773
B_0=9.4T; T=308.15K bp=24	0.3434	-0.2132	-0.1052	-0.2416	-0.1546
B_0=9.4T; T=308.15K bp=36	0.5151	-0.3198	-0.1578	-0.3624	-0.2319
B_0=11.75T; T=278.15K bp=12	0.2973	-0.1845	-0.091	-0.2091	-0.1338
B_0=11.75T; T=278.15K bp=24	0.5946	-0.369	-0.182	-0.4182	-0.2676
B_0=11.75T; T=278.15K bp=36	0.8919	-0.5535	-0.273	-0.6273	-0.4014
B_0=11.75T; T=293.15K bp=12	0.2821	-0.1751	-0.0863	-0.1984	-0.127
B_0=11.75T; T=293.15K bp=24	0.5642	-0.3502	-0.1726	-0.3968	-0.254
B_0=11.75T; T=293.15K bp=36	0.8463	-0.5253	-0.2589	-0.5952	-0.381
B_0=11.75T; T=308.15K bp=12	0.2683	-0.1665	-0.0821	-0.1887	-0.1208
B_0=11.75T; T=308.15K bp=24	0.5366	-0.333	-0.1642	-0.3774	-0.2416
B_0=11.75T; T=308.15K bp=36	0.8049	-0.4995	-0.2463	-0.5661	-0.3624
B_0=22.31T; T=278.15K bp=12	1.0717	-0.6652	-0.3281	-0.7537	-0.4824
B_0=22.31T; T=278.15K bp=24	2.1434	-1.3304	-0.6562	-1.5074	-0.9648
B_0=22.31T; T=278.15K bp=36	3.2151	-1.9956	-0.9843	-2.2611	-1.4472
B_0=22.31T; T=293.15K bp=12	1.0169	-0.6312	-0.3113	-0.7152	-0.4577

B_0=22.31T; T=293.15K bp=24	2.0338	-1.2624	-0.6226	-1.4304	-0.9154
B_0=22.31T; T=293.15K bp=36	3.0507	-1.8936	-0.9339	-2.1456	-1.3731
B_0=22.31T; T=308.15K bp=12	0.9674	-0.6004	-0.2961	-0.6804	-0.4354
B_0=22.31T; T=308.15K bp=24	1.9348	-1.2008	-0.5922	-1.3608	-0.8708
B_0=22.31T; T=308.15K bp=36	2.9022	-1.8012	-0.8883	-2.0412	-1.3062

	^{3}D H1′-H2′	^{3}D H2′-H3′	^{3}D H3′-H4′	^{3}D H4′-H5′	^{3}D H4′-H5″
B_0=28.1T; T=278.15K bp=12	1.7002	-1.0553	-0.5204	-1.1957	-0.7653
B_0=28.1T; T=278.15K bp=24	3.4004	-2.1106	-1.0408	-2.3914	-1.5306
B_0=28.1T; T=278.15K bp=36	5.1006	-3.1659	-1.5612	-3.5871	-2.2959
B_0=28.1T; T=293.15K bp=12	1.6132	-1.0013	-0.4938	-1.1345	-0.7261
B_0=28.1T; T=293.15K bp=24	3.2264	-2.0026	-0.9876	-2.269	-1.4522
B_0=28.1T; T=293.15K bp=36	4.8396	-3.0039	-1.4814	-3.4035	-2.1783
B_0=28.1T; T=308.15K bp=12	1.5347	-0.9525	-0.4698	-1.0793	-0.6908
B_0=28.1T; T=308.15K bp=24	3.0694	-1.905	-0.9396	-2.1586	-1.3816
B_0=28.1T; T=308.15K bp=36	4.6041	-2.8575	-1.4094	-3.2379	-2.0724

D)

	^{3}D H1′-C3′	^{3}D H3′-C1′	^{3}D H2′-C4′	^{3}D H4′-C2′	^{3}D H1′-C2	^{3}D H1′-C6
B_0=9.4T; T=278.15K bp=12	0.0295	0.0432	-0.0173	-0.0299	-0.0388	-0.0193
B_0=9.4T; T=278.15K bp=24	0.059	0.0864	-0.0346	-0.0598	-0.0776	-0.0386
B_0=9.4T; T=278.15K bp=36	-0.885	0.1296	-0.0519	-0.0897	-0.1164	-0.0579
B_0=9.4T; T=293.15K bp=12	0.028	0.041	-0.0164	-0.0284	-0.0368	-0.0183
B_0=9.4T; T=293.15K bp=24	0.056	0.082	-0.0328	-0.0568	-0.0736	-0.0366
B_0=9.4T; T=293.15K bp=36	0.084	0.123	-0.0492	-0.0852	-0.1104	-0.0549
B_0=9.4T; T=308.15K bp=12	0.0266	0.039	-0.0156	-0.027	-0.035	-0.0174
B_0=9.4T; T=308.15K bp=24	0.0532	0.078	-0.0312	-0.054	-0.07	-0.0348
B_0=9.4T; T=308.15K bp=36	0.0798	0.117	-0.0468	-0.081	-0.105	-0.0522
B_0=11.75T; T=278.15K bp=12	0.0461	0.0675	-0.027	-0.0468	-0.0606	-0.0302
B_0=11.75T; T=278.15K bp=24	0.0922	0.135	-0.054	-0.0936	-0.1212	-0.0604
B_0=11.75T; T=278.15K bp=36	0.1383	0.2025	-0.081	-0.1404	-0.1818	-0.0906

B_0=11.75T; T=293.15K bp=12	0.0437	0.064	-0.0256	-0.0444	-0.0575	-0.0286
B_0=11.75T; T=293.15K bp=24	0.0874	0.128	-0.0512	-0.0888	-0.115	-0.0572
B_0=11.75T; T=293.15K bp=36	0.1311	0.192	-0.0768	-0.1332	-0.1725	-0.0858
B_0=11.75T; T=308.15K bp=12	0.0416	0.0609	-0.0244	-0.0422	-0.0547	-0.0272
B_0=11.75T; T=308.15K bp=24	0.0832	0.1218	-0.0488	-0.0844	-0.1094	-0.0544
B_0=11.75T; T=308.15K bp=36	0.1248	0.1827	-0.0732	-0.1266	-0.1641	-0.0816
B_0=22.31T; T=278.15K bp=12	0.166	0.2432	-0.0974	-0.1686	-0.2185	-0.1087
B_0=22.31T; T=278.15K bp=24	0.332	0.4864	-0.1948	-0.3372	-0.437	-0.2174
B_0=22.31T; T=278.15K bp=36	0.498	0.7296	-0.2922	-0.5058	-0.6555	-0.3261
B_0=22.31T; T=293.15K bp=12	0.1575	0.2308	-0.0924	-0.1599	-0.2073	-0.1032
B_0=22.31T; T=293.15K bp=24	0.315	0.4616	-0.1848	-0.3198	-0.4146	-0.2064
B_0=22.31T; T=293.15K bp=36	0.4725	0.6924	-0.2772	-0.4797	-0.6219	-0.3096
B_0=22.31T; T=308.15K bp=12	0.1499	0.2196	-0.0879	-0.1522	-0.1973	-0.0981
B_0=22.31T; T=308.15K bp=24	0.2998	0.4392	-0.1758	-0.3044	-0.3946	-0.1962
B_0=22.31T; T=308.15K bp=36	0.4497	0.6588	-0.2367	-0.4566	-0.5919	-0.2943

	^{3}D H1′-C3′	^{3}D H3′-C1′	^{3}D H2′-C4′	^{3}D H4′-C2′	^{3}D H1′-C2	^{3}D H1′-C6
B_0=28.1T; T=278.15K bp=12	0.2634	0.3859	-0.1545	-0.2674	-0.3467	-0.1725
B_0=28.1T; T=278.15K bp=24	0.5268	0.7718	-0.309	-0.5348	-0.6934	-0.345
B_0=28.1T; T=278.15K bp=36	0.7902	1.1577	-0.4635	-0.8022	-1.0401	-0.5175
B_0=28.1T; T=293.15K bp=12	0.2499	0.3661	-0.1466	-0.2537	-0.3289	-0.1637
B_0=28.1T; T=293.15K bp=24	0.4998	0.7332	-0.2932	-0.5074	-0.6578	-0.3274
B_0=28.1T; T=293.15K bp=36	0.7497	1.0983	-0.4398	-0.7611	-0.9867	-0.4911
B_0=28.1T; T=308.15K bp=12	0.2377	0.3483	-0.1394	-0.2414	-0.3129	-0.1557
B_0=28.1T; T=308.15K bp=24	0.4754	0.6966	-0.2788	-0.4828	-0.6258	-0.3114
B_0=28.1T; T=308.15K bp=36	0.7131	1.0449	-0.4182	-0.7242	-0.9387	-0.4671

E)

	^{3}D H3′-P3′	^{3}D H5′-P5′	^{3}D H5″-P5′	^{3}D C2′-P3′	^{3}D C4′-P3′	^{3}D C4′-P5′
B_0=9.4T; T=278.15K bp=12	0.0833	-0.0192	-0.0435	0.0125	0.0064	-0.0041
B_0=9.4T; T=278.15K bp=24	0.1666	-0.0384	-0.087	0.025	0.0128	-0.0082

B_0=9.4T; T=278.15K bp=36	0.2499	-0.0576	-0.1305	0.0375	0.0192	-0.0123
B_0=9.4T; T=293.15K bp=12	0.079	-0.0182	-0.0412	0.0119	0.0061	-0.0039
B_0=9.4T; T=293.15K bp=24	0.158	-0.0364	-0.0824	0.0238	0.0122	-0.0078
B_0=9.4T; T=293.15K bp=36	0.237	-0.0546	-0.1236	0.0357	0.0183	-0.0117
B_0=9.4T; T=308.15K bp=12	0.0752	-0.0173	-0.0392	0.0113	0.0058	-0.0037
B_0=9.4T; T=308.15K bp=24	0.1504	-0.0346	-0.0784	0.0226	0.0116	-0.0074
B_0=9.4T; T=308.15K bp=36	0.2256	-0.0519	-0.1176	0.0339	0.0174	-0.0111
B_0=11.75T; T=278.15K bp=12	0.1301	-0.03	-0.0679	0.0195	0.0101	-0.0064
B_0=11.75T; T=278.15K bp=24	0.2602	-0.06	-0.1358	0.039	0.0202	-0.0128
B_0=11.75T; T=278.15K bp=36	0.3903	-0.09	-0.2037	0.0585	0.0303	-0.0192
B_0=11.75T; T=293.15K bp=12	0.1235	-0.0284	-0.0644	0.0185	0.0096	-0.0061
B_0=11.75T; T=293.15K bp=24	0.247	-0.0568	-0.1288	0.037	0.0192	-0.0122
B_0=11.75T; T=293.15K bp=36	0.3705	-0.0852	-0.1932	0.0555	0.0288	-0.0183
B_0=11.75T; T=308.15K bp=12	0.1174	-0.027	-0.0613	0.0176	0.0091	-0.0058
B_0=11.75T; T=308.15K bp=24	0.2348	-0.054	-0.1226	0.0352	0.0182	-0.0116
B_0=11.75T; T=308.15K bp=36	0.3522	-0.081	-0.1839	0.0528	0.0273	-0.0174
B_0=17.6T; T=278.15K bp=12	0.2919	-0.0672	-0.1523	0.0438	0.0226	-0.0144
B_0=17.6T; T=278.15K bp=24	0.5838	-0.1344	-0.3046	0.0876	0.0452	-0.0288
B_0=17.6T; T=278.15K bp=36	0.8757	-0.2016	-0.4569	0.1314	0.0678	-0.0432
B_0=17.6T; T=293.15K bp=12	0.2770	-0.0638	-0.1445	0.0416	0.0214	-0.0137
B_0=17.6T; T=293.15K bp=24	0.5540	-0.1276	-0.2890	0.0832	0.0428	-0.0274
B_0=17.6T; T=293.15K bp=36	0.8310	-0.1914	-0.4335	0.1248	0.0642	-0.0411
B_0=17.6T; T=308.15K bp=12	0.2635	-0.0607	-0.1375	0.0396	0.0204	-0.0130
B_0=17.6T; T=308.15K bp=24	0.5270	-0.1214	-0.2750	0.0792	0.0408	-0.0260
B_0=17.6T; T=308.15K bp=36	0.7905	-0.1821	-0.4125	0.1188	0.0612	-0.0390

Table S3: Calculated magnetic field induced dipolar couplings $^{1}D_{CH}$ [Hz] (A), $^{2}D_{CH}$ (B), $^{3}D_{HH}$ (C), $^{3}D_{HC}$ (D), $^{3}D_{HP/CP}$ (E) for residue Gua10 in canonical 12, 24, and 36 bp B-DNA as a function of magnetic field strength B_0 (9.4T, 11.75T, 22.31T and 28.1T) and temperature T (278.15K, 293.15K, 308.15K).

A)

	^{1}D C1′-H1′	^{1}D C2′-H2′	^{1}D C3′-H3′	^{1}D C5′-H5′	^{1}D C5′-H5′′
B_0=9.4T; T=278.15K bp=12	-0.4644	-0.5936	-0.54	0.3774	-0.3246
B_0=9.4T; T=278.15K bp=24	-0.9288	-1.1872	-1.08	0.7548	-0.6492
B_0=9.4T; T=278.15K bp=36	-1.3932	-1.7808	-1.62	1.1322	-0.9738
B_0=9.4T; T=293.15K bp=12	-0.4406	-0.5632	-0.5124	0.3581	-0.308
B_0=9.4T; T=293.15K bp=24	-0.8812	-1.1264	-1.0248	0.7162	-0.616
B_0=9.4T; T=293.15K bp=36	-1.3218	-1.6896	-1.5372	1.0743	-0.924
B_0=9.4T; T=308.15K bp=12	-0.4192	-0.5358	-0.4874	0.3407	-0.293
B_0=9.4T; T=308.15K bp=24	-0.8384	-1.0716	-0.9748	0.6814	-0.586
B_0=9.4T; T=308.15K bp=36	-1.2576	-1.6074	-1.4622	1.0221	-0.879
B_0=11.75T; T=278.15K bp=12	-0.7256	-0.9274	-0.8437	0.5898	-0.5072
B_0=11.75T; T=278.15K bp=24	-1.4512	-1.8548	-1.6874	1.1796	-1.0144
B_0=11.75T; T=278.15K bp=36	-2.1768	-2.7822	-2.5311	1.7694	-1.5216
B_0=11.75T; T=293.15K bp=12	-0.6885	-0.88	-0.8006	0.5596	-0.4813
B_0=11.75T; T=293.15K bp=24	-1.377	-1.76	-1.6012	1.1192	-0.9626
B_0=11.75T; T=293.15K bp=36	-2.0655	-2.64	-2.4018	1.6788	-1.4439
B_0=11.75T; T=308.15K bp=12	-0.655	-0.8371	-0.7616	0.5323	-0.4578
B_0=11.75T; T=308.15K bp=24	-1.31	-1.6742	-1.5232	1.0646	-0.9156
B_0=11.75T; T=308.15K bp=36	-1.965	-2.5113	-2.2848	1.5969	-1.3734
B_0=22.31T; T=278.15K bp=12	-2.6159	-3.3435	-3.0418	2.1262	-1.8286
B_0=22.31T; T=278.15K bp=24	-5.2318	-6.687	-6.0836	4.2524	-3.6572
B_0=22.31T; T=278.15K bp=36	-7.8477	-10.0305	-9.1254	6.3786	-5.4858
B_0=22.31T; T=293.15K bp=12	-2.4821	-3.1724	-2.8862	2.0174	-1.7351
B_0=22.31T; T=293.15K bp=24	-4.9642	-6.3448	-5.7724	4.0348	-3.4702
B_0=22.31T; T=293.15K bp=36	-7.4463	-9.5172	-8.6586	6.0522	-5.2053
B_0=22.31T; T=308.15K	-2.3613	-3.018	-2.7457	1.9192	-1.6506

bp=12					
B_0=22.31T; T=308.15K bp=24	-4.7226	-6.036	-5.4914	3.8384	3.3012
	^{1}D C1′-H1′	^{1}D C2′-H2′	^{1}D C3′-H3′	^{1}D C5′-H5′	^{1}D C5′-H5″
B_0=22.31T; T=308.15K bp=36	-7.0839	-9.054	-8.2371	5.7576	-4.9518
B_0=28.1T; T=278.15K bp=12	-4.1499	-5.3042	-4.8255	3.373	-2.9009
B_0=28.1T; T=278.15K bp=24	-8.2998	-10.6084	-9.651	6.746	-5.8018
B_0=28.1T; T=278.15K bp=36	-12.4497	-15.9126	-14.4765	10.119	-8.7027
B_0=28.1T; T=293.15K bp=12	-3.9376	-5.0327	-4.5786	3.2004	-2.7525
B_0=28.1T; T=293.15K bp=24	-7.8752	-10.0654	-9.1572	6.4008	-5.505
B_0=28.1T; T=293.15K bp=36	-11.8128	-15.0981	-13.7358	9.6012	-8.2575
B_0=28.1T; T=308.15K bp=12	-3.7459	-4.7878	-4.3557	3.0446	-2.6185
B_0=28.1T; T=308.15K bp=24	-7.4918	-9.5756	-8.7114	6.0892	-5.237
B_0=28.1T; T=308.15K bp=36	-11.2377	-14.3634	-13.0671	9.1338	-7.8555

B)

	^{2}D C1′-H2′	^{2}D C2′-H3′	^{2}D C3′-H2′	^{2}D C4′-H3′
B_0=9.4T; T=278.15K bp=12	-0.0333	-0.0591	-0.0391	0.0558
B_0=9.4T; T=278.15K bp=24	-0.0666	-0.1182	-0.0782	0.1116
B_0=9.4T; T=278.15K bp=36	-0.0999	-0.1773	-0.1173	0.1674
B_0=9.4T; T=293.15K bp=12	-0.0316	-0.0561	-0.0371	0.053
B_0=9.4T; T=293.15K bp=24	-0.0632	-0.1122	-0.0742	0.106
B_0=9.4T; T=293.15K bp=36	-0.0948	-0.1683	-0.1113	0.159
B_0=9.4T; T=308.15K bp=12	-0.0301	-0.0533	-0.0353	0.0504
B_0=9.4T; T=308.15K bp=24	-0.0602	-0.1066	-0.0706	0.1008
B_0=9.4T; T=308.15K bp=36	-0.0903	-0.1599	-0.1059	0.1512
B_0=11.75T; T=278.15K bp=12	-0.0521	-0.0923	-0.0611	0.0872
B_0=11.75T; T=278.15K bp=24	-0.1042	-0.1846	-0.1222	0.1744
B_0=11.75T; T=278.15K bp=36	-0.1563	-0.2769	-0.1833	0.2616
B_0=11.75T; T=293.15K bp=12	-0.0494	-0.0876	-0.058	0.0828
B_0=11.75T; T=293.15K bp=24	-0.0988	-0.1752	-0.116	0.1656

B_0=11.75T; T=293.15K bp=36	-0.1482	-0.2628	-0.174	0.2484
B_0=11.75T; T=308.15K bp=12	-0.047	-0.0833	-0.0551	0.0788
B_0=11.75T; T=308.15K bp=24	-0.094	-0.1666	-0.1102	0.1576
B_0=11.75T; T=308.15K bp=36	-0.141	-0.2499	-0.1653	0.2364
B_0=22.31T; T=278.15K bp=12	-0.1877	-0.3328	-0.2203	0.3145
B_0=22.31T; T=278.15K bp=24	-0.3754	-0.6656	-0.4406	0.629
B_0=22.31T; T=278.15K bp=36	-0.5631	-0.9984	-0.6609	0.9435
B_0=22.31T; T=293.15K bp=12	-0.1781	-0.3158	-0.209	0.2984
B_0=22.31T; T=293.15K bp=24	-0.3562	-0.6316	-0.418	0.5968
B_0=22.31T; T=293.15K bp=36	-0.5343	-0.9474	-0.627	0.8952
B_0=22.31T; T=308.15K bp=12	-0.1695	-0.3004	-0.1988	0.2839
B_0=22.31T; T=308.15K bp=24	-0.339	-0.6008	-0.3976	0.5678
B_0=22.31T; T=308.15K bp=36	-0.5085	-0.9012	-0.5964	0.8517
	^{2}D C1′-H2′	^{2}D C2′-H3′	^{2}D C3′-H2′	^{2}D C4′-H3′
B_0=28.1T; T=278.15K bp=12	-0.2978	-0.528	-0.3494	0.499
B_0=28.1T; T=278.15K bp=24	-0.5956	-1.056	-0.6988	0.998
B_0=28.1T; T=278.15K bp=36	-0.8934	-1.584	-1.0482	1.497
B_0=28.1T; T=293.15K bp=12	-0.2826	-0.501	-0.3315	0.4734
B_0=28.1T; T=293.15K bp=24	-0.5652	-1.002	-0.663	0.9468
B_0=28.1T; T=293.15K bp=36	-0.8478	-1.053	-0.9945	1.4202
B_0=28.1T; T=308.15K bp=12	-0.2688	-0.4766	-0.3154	0.4504
B_0=28.1T; T=308.15K bp=24	-0.5376	-0.9532	-0.6308	0.9008
B_0=28.1T; T=308.15K bp=36	-0.8064	-1.4298	-0.9462	1.3512

C)

	^{3}D H1′-H2′	^{3}D H1′-H2″	^{3}D H2′-H3′	^{3}D H3′-H4′	^{3}D H4′-H5′	^{3}D H4′-H5″
B_0=9.4T; T=278.15K bp=12	-0.0938	0.067	-0.152	0.1435	-0.091	-0.2195
B_0=9.4T; T=278.15K bp=24	-0.1876	0.134	-0.304	0.287	-0.182	-0.439
B_0=9.4T; T=278.15K bp=36	-0.2814	0.201	-0.456	0.4305	-0.273	-0.6585
B_0=9.4T; T=293.15K bp=12	-0.089	0.0636	-0.1443	0.1361	-0.0863	-0.2083

B_0=9.4T; T=293.15K bp=24	-0.178	0.1272	-0.2886	0.2722	-0.1726	-0.4166
B_0=9.4T; T=293.15K bp=36	-0.267	0.1908	-0.4329	0.4083	-0.2589	-0.6249
B_0=9.4T; T=308.15K bp=12	-0.0847	0.0605	-0.1372	0.1295	-0.0821	-0.1981
B_0=9.4T; T=308.15K bp=24	-0.1694	0.121	-0.2744	0.259	-0.1642	-0.3962
B_0=9.4T; T=308.15K bp=36	-0.2541	0.1815	-0.4116	-0.3885	-0.2463	-0.5943
B_0=11.75T; T=278.15K bp=12	-0.1466	0.1047	-0.2376	0.2241	-0.1422	-0.3429
B_0=11.75T; T=278.15K bp=24	-0.2932	0.2094	-0.4752	0.4482	-0.2844	-0.6858
B_0=11.75T; T=278.15K bp=36	-0.4398	0.3141	-0.7128	0.6723	-0.4266	-1.0287
B_0=11.75T; T=293.15K bp=12	-0.3191	0.0994	-0.2254	0.2127	-0.1349	-0.3254
B_0=11.75T; T=293.15K bp=24	-0.2782	0.1988	-0.4508	0.4254	-0.2698	-0.6508
B_0=11.75T; T=293.15K bp=36	-0.4173	0.2982	-0.6762	0.6381	-0.4047	-0.9762
B_0=11.75T; T=308.15K bp=12	-0.1324	0.0945	-0.2144	0.2023	-0.2183	-0.3096
B_0=11.75T; T=308.15K bp=24	-0.2648	0.189	-0.4288	0.4046	-0.2566	-0.6192
B_0=11.75T; T=308.15K bp=36	-0.3972	0.2835	-0.6432	0.6069	-0.3849	-0.9288
B_0=22.31T; T=278.15K bp=12	-0.5286	0.3776	-0.8565	0.8081	-0.5125	-1.2364
B_0=22.31T; T=278.15K bp=24	-1.0572	0.7552	-1.713	1.6162	-1.025	-2.4728
B_0=22.31T; T=278.15K bp=36	-1.5858	1.1328	-2.5695	2.4243	-1.5375	-3.7092
B_0=22.31T; T=293.15K bp=12	-0.5016	0.3583	-0.8126	0.7667	-0.4863	-1.1731
B_0=22.31T; T=293.15K bp=24	-1.0032	0.7166	-1.6252	1.5334	-0.9726	-2.3462
B_0=22.31T; T=293.15K bp=36	-1.5048	1.0749	-2.4378	2.3001	-1.4589	-3.5193
B_0=22.31T; T=308.15K bp=12	-0.4772	0.3409	-0.7731	0.7294	-0.4626	-1.116
B_0=22.31T; T=308.15K bp=24	-0.9544	0.6818	-1.5462	1.4588	-0.9252	-2.232
B_0=22.31T; T=308.15K bp=36	-1.4316	1.0227	-2.3193	2.1882	-1.3878	-3.348

	^{3}D H1′-H2′	^{3}D H1′-H2″	^{3}D H2′-H3′	^{3}D H3′-H4′	^{3}D H4′-H5′	^{3}D H4′-H5″
B_0=28.1T; T=278.15K bp=12	-0.8386	0.599	-1.3587	1.2819	-0.8131	-1.9614
B_0=28.1T; T=278.15K bp=24	-1.6772	1.198	-2.7174	2.5638	-1.6262	-3.9228
B_0=28.1T; T=278.15K bp=36	-2.5158	1.797	-4.0761	3.8457	-2.4393	-5.8842
B_0=28.1T; T=293.15K bp=12	-0.7957	0.5684	-1.2892	1.2163	-0.7715	-1.861
B_0=28.1T; T=293.15K	-1.5914	1.1368	-2.5784	2.4326	1.543	-3.722

bp=24						
B_0=28.1T; T=293.15K bp=36	-2.3871	1.7052	-3.8676	3.6489	-2.3145	-5.583
B_0=28.1T; T=308.15K bp=12	-0.757	0.5407	-1.2264	1.1571	-0.7339	-1.7704
B_0=28.1T; T=308.15K bp=24	-1.514	1.0814	-2.4528	2.3142	-1.4678	-3.5408
B_0=28.1T; T=308.15K bp=36	-2.271	1.6221	-3.6792	3.4713	-2.2017	-5.3112

D)

	^{3}D H1′-C3′	^{3}D H3′-C1′	^{3}D H2′-C4′	^{3}D H4′-C2′	^{3}D H1′-C4	^{3}D H1′-C8
B_0=9.4T; T=278.15K bp=12	-0.0414	-0.0215	0.0268	0.0236	-0.0411	-0.0203
B_0=9.4T; T=278.15K bp=24	-0.0828	-0.043	0.0536	0.0472	-0.0822	-0.0406
B_0=9.4T; T=278.15K bp=36	-0.1242	-0.0645	0.0804	0.0708	-0.1233	-0.609
B_0=9.4T; T=293.15K bp=12	-0.0393	-0.0204	0.0254	0.0224	-0.039	-0.0193
B_0=9.4T; T=293.15K bp=24	-0.0786	-0.0408	0.0508	0.0448	-0.078	-0.0386
B_0=9.4T; T=293.15K bp=36	-0.1179	-0.0612	0.0762	0.0672	-0.117	-0.0579
B_0=9.4T; T=308.15K bp=12	-0.0374	-0.0194	0.0242	0.0213	-0.0371	-0.0183
B_0=9.4T; T=308.15K bp=24	-0.0748	-0.0388	0.0484	0.0426	-0.0742	-0.0366
B_0=9.4T; T=308.15K bp=36	-0.1122	-0.0582	0.0726	0.0639	-0.113	-0.0549
B_0=11.75T; T=278.15K bp=12	-0.0674	-0.0336	0.0418	0.0368	-0.0642	-0.0317
B_0=11.75T; T=278.15K bp=24	-0.1348	-0.0672	0.0836	0.0736	-0.1284	-0.0634
B_0=11.75T; T=278.15K bp=36	-0.2022	-0.1008	0.1254	0.1104	-0.1926	-0.0951
B_0=11.75T; T=293.15K bp=12	-0.0614	-0.0319	0.0397	0.0349	-0.0609	-0.0301
B_0=11.75T; T=293.15K bp=24	-0.1228	-0.0638	0.0794	0.0698	-0.1218	-0.0602
B_0=11.75T; T=293.15K bp=36	-0.1842	-0.0957	0.1191	0.1047	-0.1827	-0.0903
B_0=11.75T; T=308.15K bp=12	-0.0584	-0.0303	0.0378	0.0332	-0.0579	-0.0286
B_0=11.75T; T=308.15K bp=24	-0.1168	-0.0606	0.0756	0.0664	-0.1158	-0.0572
B_0=11.75T; T=308.15K bp=36	-0.1752	-0.0909	0.1134	0.0996	-0.1737	-0.0858
B_0=22.31T; T=278.15K bp=12	-0.2332	-0.1211	0.1508	0.1327	-0.2314	-0.1143
B_0=22.31T; T=278.15K bp=24	-0.4664	-0.2422	0.3016	0.2654	-0.4628	-0.2286
B_0=22.31T; T=278.15K bp=36	-0.6996	-0.3633	0.4524	0.3981	-0.6942	-0.3429
B_0=22.31T; T=293.15K bp=12	-0.2213	-0.1149	0.1431	0.1259	-0.2196	-0.1085
B_0=22.31T; T=293.15K	-0.4426	-0.2298	0.2862	0.2518	-0.4392	-0.217

bp=24						
B_0=22.31T; T=293.15K bp=36	-0.6639	-0.3447	0.4293	0.3777	-0.6588	-0.3255
B_0=22.31T; T=308.15K bp=12	-0.2105	-0.1093	0.1361	0.1198	-0.2089	-0.1032
B_0=22.31T; T=308.15K bp=24	-0.421	-0.2186	0.2722	0.2396	-0.4178	-0.2064
B_0=22.31T; T=308.15K bp=36	-0.6315	-0.3279	0.4083	0.3594	-0.6267	-0.3096

	^{3}D H1′-C3′	^{3}D H3′-C1′	^{3}D H2′-C4′	^{3}D H4′-C2′	^{3}D H1′-C4	^{3}D H1′-C8
B_0=28.1T; T=278.15K bp=12	-0.37	-0.1921	0.2392	0.2106	-0.3671	-0.1814
B_0=28.1T; T=278.15K bp=24	-0.74	-0.3842	0.4784	0.4212	-0.7342	-0.3628
B_0=28.1T; T=278.15K bp=36	-1.11	-0.5763	0.7176	0.6318	-1.1013	-0.5442
B_0=28.1T; T=293.15K bp=12	-0.351	-0.1822	0.227	0.1998	-0.3484	-0.1721
B_0=28.1T; T=293.15K bp=24	-0.702	-0.3644	0.454	0.3996	-0.6968	-0.3442
B_0=28.1T; T=293.15K bp=36	-1.053	-0.5466	0.681	0.5994	-1.0452	-0.5163
B_0=28.1T; T=308.15K bp=12	-0.334	-0.1734	0.2159	0.1901	-0.3314	0.1637
B_0=28.1T; T=308.15K bp=24	-0.668	-0.3468	0.4318	0.3802	-0.6628	0.3274
B_0=28.1T; T=308.15K bp=36	-1.002	-0.5202	0.6477	0.5703	-0.9942	0.4911

E)

	^{3}D H3′-P3′	^{3}D H5′-P5′	^{3}D H5″-P5′	^{3}D C2′-P3′	^{3}D C4′-P3′	^{3}D C4′-P5′
B_0=9.4T; T=278.15K bp=12	-0.0298	-0.0315	-0.0316	-0.0066	0.0038	-0.0056
B_0=9.4T; T=278.15K bp=24	-0.0596	-0.063	-0.0632	-0.0132	0.0076	-0.0112
B_0=9.4T; T=278.15K bp=36	-0.0894	-0.0945	-0.0948	-0.0198	0.0114	-0.0168
B_0=9.4T; T=293.15K bp=12	-0.0283	-0.0299	-0.03	-0.0062	0.0036	-0.0053
B_0=9.4T; T=293.15K bp=24	-0.0566	-0.0598	-0.06	-0.0124	0.0072	-0.0106
B_0=9.4T; T=293.15K bp=36	-0.0849	-0.0897	-0.09	-0.0186	0.0108	-0.0159
B_0=9.4T; T=308.15K bp=12	-0.0269	-0.0284	-0.0285	-0.0059	0.0034	-0.0051
B_0=9.4T; T=308.15K bp=24	-0.0538	-0.0568	-0.057	-0.0118	0.0068	-0.0102
B_0=9.4T; T=308.15K bp=36	-0.0807	-0.0852	-0.0855	-0.0177	0.0102	-0.0153
B_0=11.75T; T=278.15K bp=12	-0.0466	-0.0492	-0.0494	-0.0103	0.0059	-0.0087
B_0=11.75T; T=278.15K bp=24	-0.0932	-0.0984	-0.0988	-0.0206	0.0118	-0.0174
B_0=11.75T; T=278.15K bp=36	-0.1398	-0.1476	-0.1482	-0.0309	0.0177	-0.0261

B_0=11.75T; T=293.15K bp=12	-0.0442	-0.0466	-0.0469	-0.0097	0.056	-0.0083
B_0=11.75T; T=293.15K bp=24	-0.0884	-0.0932	-0.0938	-0.0194	0.112	-0.0166
B_0=11.75T; T=293.15K bp=36	-0.1326	-0.1398	-0.1407	-0.0291	0.168	-0.0249
B_0=11.75T; T=308.15K bp=12	-0.0421	-0.0444	-0.0446	-0.0093	0.0054	-0.0079
B_0=11.75T; T=308.15K bp=24	-0.0842	-0.0888	-0.0892	-0.0186	0.0108	-0.0158
B_0=11.75T; T=308.15K bp=36	-0.1263	-0.1332	-0.1338	-0.0279	0.0162	-0.0237
B_0=17.6T; T=278.15K bp=12	-0.1046	-0.1103	-0.1108	-0.0230	0.0133	-0.0196
B_0=17.6T; T=278.15K bp=24	-0.2092	-0.2206	-0.2216	-0.0460	0.0266	-0.0392
B_0=17.6T; T=278.15K bp=36	-0.3138	-0.3309	-0.3324	-0.0690	0.0399	-0.0588
B_0=17.6T; T=293.15K bp=12	-0.0993	-0.1047	-0.1051	-0.0218	0.0127	-0.0186
B_0=17.6T; T=293.15K bp=24	-0.1986	-0.2094	-0.2102	-0.0436	0.0254	-0.0372
B_0=17.6T; T=293.15K bp=36	-0.2979	-0.3141	-0.3153	-0.0654	0.0381	-0.0558
B_0=17.6T; T=308.15K bp=12	-0.0944	-0.0996	-0.1000	-0.0208	0.0120	-0.0177
B_0=17.6T; T=308.15K bp=24	-0.1888	-0.1992	-0.2000	-0.0416	0.0240	-0.0354
B_0=17.6T; T=308.15K bp=36	-0.2832	-0.2988	-0.3000	-0.0624	0.0360	-0.0531
